# Awareness of Prediabetes and Diabetes among Persons with Clinical Depression

**DOI:** 10.1155/2015/839152

**Published:** 2015-04-28

**Authors:** Mary Rosedale, Shiela M. Strauss, Candice Knight, Dolores Malaspina

**Affiliations:** ^1^New York University College of Nursing, New York, NY 10010, USA; ^2^NYU Langone Medical Center, New York, NY 10016, USA

## Abstract

*Background*. Major depressive disorder (MDD) is highly comorbid with diabetes, a relationship underappreciated by clinicians.* Purpose*. Examine the proportion of nonpregnant individuals ≥20 years with MDD and elevated glucose and the demographic and clinical characteristics associated with unrecognized elevated glucose.* Methods*. 14,373 subjects who participated in the National Health and Nutrition Examination Survey (2007–2012) completed the PHQ-9 depression screen and had hemoglobin A1C (HbA1c) measured. PHQ-9 scores ≥10 and HbA1c scores ≥5.7% were defined as MDD and elevated HbA1c, respectively. Data were analyzed using complex survey sampling software.* Results*. 38.4% of the sample with MDD had elevated HbA1c readings. Compared with nondepressed subjects, they were significantly more likely to have elevated glucose readings (*P* = 0.003) and to be aware of their elevated glucose levels if they had a higher body mass index, family history of diabetes, more doctor visits in the past year, a usual care source, health insurance, or were taking hypertension or hypercholesterolemia medications.* Conclusions*. Many adults with MDD have elevated HbA1c levels, have never been advised of elevated HbA1c, have not received diabetes screening, and have minimal contact with a healthcare provider. Additional opportunities for diabetes risk screening in people with MDD are needed.

## 1. Introduction

An estimated 7 million people with diabetes in the United States were thought to have remained undiagnosed in 2010 [1]. This is of great concern because as many as 25% of people with a new diagnosis of diabetes have already developed diabetic retinopathy or microalbuminuria, suggesting that there is a 4–7-year gap between the diagnosis of type 2 diabetes and its onset [2–4]. In fact, micro- and macrovascular complications are sometimes present, even in prediabetes (i.e., impaired glucose tolerance or impaired fasting glucose), a condition that often progresses to diabetes in the absence of lifestyle changes in diet, weight loss, and physical activity [5]. Importantly, early detection of elevated glucose values, whether in the prediabetes or diabetes ranges, could identify these complications at an earlier stage [6–14]. Good management of blood glucose levels, blood pressure and cholesterol control, and smoking cessation support could reduce diabetes-related symptoms and its acute and chronic complications [15].

Notably, the Centers for Disease Control and Prevention (CDC) in the United States indicate that depression is associated with a 60% increased risk of developing diabetes [1]. Consistent with the CDC report, many studies that have examined the relationship between depression and the subsequent development of diabetes have determined that depression is a risk factor for incident prediabetes and diabetes [16–25]. In view of this elevated risk, early identification of prediabetes and diabetes in persons with depression is especially important, so that their health can be preserved to the greatest extent possible. Little is known, however, about the degree to which persons with clinical depression and elevated glucose are aware that they have prediabetes or diabetes, or the specific sociodemographic or other characteristics of these persons.

To fill this gap in knowledge, we analyzed data from a nationally representative sample of persons in the United States from 2007 to 2012. We first compared various sociodemographic characteristics, health-care access or use characteristics, and other factors among nonpregnant persons at least 20 years of age according to whether they had glycemic values in the normal or elevated range. We then determined the proportion of nonpregnant individuals 20 years of age or older with clinical depression and elevated glycemic values irrespective of whether or not they were aware that they had diabetes or prediabetes. Finally, we divided the subgroup of persons who had elevated glycemic values according to whether or not they had been told by a health provider that they had diabetes, had prediabetes, or were borderline for diabetes. We made comparisons in this regard across various sociodemographic characteristics, health-care access or use characteristics, and other factors. Identifying the characteristics of adults with clinical depression who are unaware of their elevated glycemic levels will enable the development of strategies that target the individual needs of depressed persons with prediabetes and diabetes.

## 2. Methods 

To perform the analyses, we used data from the National Health and Nutrition Examination Survey (NHANES) 2007–2012. NHANES is conducted by the National Center for Health Statistics, Centers for Disease Control and Prevention, to assess the health status of a representative sample of civilian, noninstitutionalized adults, and children in the United States, through interviews and direct examinations. Notably, sample selection for NHANES does not use random sampling. Rather, a complex, multistage, probability sampling design is used to select participants. Oversampling of certain population subgroups is done to increase the reliability and precision of health status indicator estimates for these groups. To account for its sampling design, NHANES provides sampling weights and sample design variables for use in its analyses so that unbiased estimates can be obtained and so that significance levels are not overstated. The sample weight for each person is a measure of the number of people in the population represented by that sample person in NHANES, reflecting the unequal probability of selection, nonresponse adjustment, and adjustment to independent population controls. The sample weights are then used to produce an unbiased national estimate and enable the reporting of results using the sample so that they represent the United States Census civilian noninstitutionalized population. A full description of the design of NHANES is available on the NHANES website: http://www.cdc.gov/nchs/data/nhanes.htm [26].

**Figure 1:**
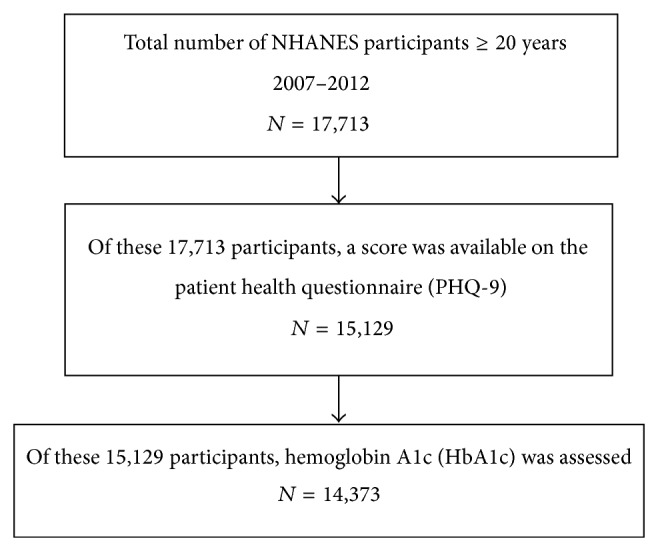
Study subjects.

As can be seen in Figure 1, the NHANES 2007-2008, 2009-2010, and 2011-2012 datasets comprised a total sample of 17,713 individuals, 20 years of age or older. Depression was assessed using the Patient Health Questionnaire-9 (PHQ-9), an instrument that was administered in English or Spanish to NHANES participants 12 years of age or older by trained interviewers. The PHQ-9 is the depression module of the Patient Health Questionnaire (PHQ), a PRIME-MD diagnostic instrument for common mental disorders and is an evidence-based, depression screening tool with demonstrated excellent reliability and validity in primary care and obstetrics-gynecology clinics [27–31]. The PHQ-9 scores assess each of the 9 DSM-IV (and newly updated DSM-5) criteria for major depressive disorder as “0” (not at all) to “3” (nearly every day). Although the criteria require a score of only 5 to meet the diagnostic threshold, a higher score of 10 is generally considered clinically significant depression and the benchmark to warrant depression treatment [32]. Consistent with NHANES and DSM-IV and DSM-5, we categorized those with PHQ-9 scores <10 as not depressed and those with scores of 10 or higher as clinically depressed [33]. A total of 15,129 of the 17,713 individuals in the NHANES 2007–2012 datasets who were 20 years of age or older had PHQ-9 scores that enabled this categorization.

To assess elevated glycemic values in the laboratory, NHANES conducted venous blood draws in participants ≥12 years to measure hemoglobin A1c (HbA1c). We applied American Diabetes Association criteria to determine if these measures were in the normal range (i.e., <5.7% [39 mmol/mol]) or in the prediabetes or diabetes ranges (i.e., ≥5.7% [39 mmol/mol]). A total of 14,373 of the 15,129 individuals in the NHANES 2007–2012 datasets who were 20 years of age or older, not pregnant, and classified according to clinical depression also had HbA1c levels assessed.

NHANES participants also completed a questionnaire that contained items of special relevance to our analyses. These included various sociodemographic characteristics, health-care access or use characteristics, and other factors, as had been considered in the CDC analysis concerning awareness of prediabetes in the total population of individuals at least 20 years of age [34]. Sociodemographic characteristics included age group, race/ethnicity, sex, and education level. Health-care access or use characteristics included having any health insurance or other health-care coverage at the time of the interview, number of visits to doctors in the past year, and having a usual source of care (i.e., having a place usually visited for care that was a doctor's office or clinic as opposed to not having such a place or using a hospital outpatient or emergency department). Other characteristics examined included family history of diabetes, physical inactivity (i.e., spending < 30 minutes in moderate or vigorous activity at work and/or during leisure time each day), reported current use of medication for hypertension and/or hypercholesterolemia, and body mass index (BMI) classified as normal weight (BMI < 25.0 kg/m^2^), overweight (BMI 25.0–29.9 kg/m^2^), or obese (BMI ≥ 30.0 kg/m^2^).

Study sample participants were also classified as being aware of having prediabetes or diabetes if they either indicated that (1), other than during pregnancy, they had ever been told by a doctor or health professional that they had diabetes or were borderline for diabetes, or (2) they had ever been told by a doctor that they had prediabetes, borderline diabetes, impaired fasting glucose, or impaired glucose tolerance or that their blood sugar was higher than normal but not high enough to be called diabetes or sugar diabetes.

Our analyses were performed using complex survey sampling software from IBM PASW. In our analyses, we followed the analytic guidelines provided by NHANES, available at http://www.cdc.gov/nchs/data/nhanes/nhanes_03_04/nhanes_analytic_guidelines_dec_2005.pdf, and used the examination weights provided in order to extrapolate the analyses to the entire civilian, noninstitutionalized, nonpregnant adult United States population ≥ 20 years. The analyses were both descriptive (i.e., frequencies) and inferential, the latter involving chi-square tests, with the Likelihood Ratio test used to examine statistical significance. To examine statistical significance for the chi-square tests (with *P* = 0.05 considered statistically significant), IBM PASW uses the adjusted F and its degrees of freedom, with the adjusted F a variant of the second-order Rao-Scott adjusted chi-square statistic. NHANES is among the sources of public use data approved by the University Committee on Activities Involving Human Subjects at New York University, thereby allowing New York University investigators to use the database without review and approval by that committee.

## 3. Results

*Differences between Those with Normal and Elevated HbA1c Values (in the Prediabetes and Diabetes Ranges)*. As can be seen in Table 1, when the 14,373 individuals in the NHANES 2007–2012 datasets who were 20 years of age or older, not pregnant, and were classified according to normal versus elevated HbA1c values (HbA1c < 5.7% [39 mmol/mol] versus HbA1c ≥ 5.7% [39 mmol/mol]), they differed significantly according to age (*P* < 0.001). When extrapolated to the civilian, noninstitutionalized, and nonpregnant United States population ≥ 20 years of age, 57% of persons with normal range HbA1c were under 45 years, and only 5.5% were at least 65 years, while 59.5% of those with elevated HbA1c were between 45 and 64 years and the remainder were approximately equally divided between those under 45 years and those at least 65 years (22% and 18.5%, resp.). In addition, while those with elevated HbA1c levels were approximately evenly divided among those who did not complete high school, those who had a high school diploma, and those educated beyond high school (36.7%, 29.4%, and 33.9%, resp.), 50.4% of persons with a normal HbA1c had more than a high school education (*P* = 0.002). Differences were also seen for persons regarding their BMI: those with normal range HbA1c were about evenly divided between those who had normal weight, were overweight, and were obese (31.8%, 33.1%, and 35.1%, resp.), while 60.9% of persons with elevated HbA1c were obese (*P* < 0.001). In addition, as compared with persons with normal HbA1c, those with elevated HbA1c levels were more likely to have a family history of diabetes (54.8% versus 43.8%; *P* = 0.004), to be physically inactive (63.1% versus 44.5%; *P* < 0.001), and to take medication for hypertension and/or hypercholesterolemia (52.8% versus 19.4%; *P* < 0.001). Persons with elevated HbA1c levels were also more likely to have a greater number of physician visits in the past year (16.9% had <2 visits, 17.1% had 2-3, and 65.9% had ≥4) than those with HbA1c levels in the normal range (25.8% had <2 visits, 21.7% had 2-3, and 52.5% had ≥4; *P* < 0.001). However, there were no statistically significant sex or race/ethnicity differences between the two groups of persons according to whether they had normal range or elevated range HbA1c values, nor were there differences between these groups in terms of whether they had health insurance coverage or a usual source of health care.

Of the 14,373 individuals in the NHANES 2007–2012 datasets who were 20 years of age or older, were not pregnant, had HbA1c levels assessed, and were classified according to clinical depression (defined as a PHQ-9 score ≥ 10), 1355 were clinically depressed and 594 were both clinically depressed and had elevated HbA1c. When extrapolated to the civilian, noninstitutionalized nonpregnant United States population at least 20 years of age, 7.9% were clinically depressed and 3.0% were both clinically depressed and had elevated HbA1c.

*Relationship between Clinical Depression and Elevated Glucose*. We next used data from NHANES 2007–2012 to determine the relationship between clinical depression and normal versus elevated HbA1c values for nonpregnant adults ≥ 20 years extrapolated to the civilian, noninstitutionalized United States population (Table 2). Depression was significantly associated with glucose levels (*P* = 0.003). As can be seen in Table 2, while 32.3% of those who scored in the nonclinically depressed range on the PHQ-9 had elevated HbA1c levels, this was the case for 38.4% of those who scored in the clinically depressed range.

*Relationship between Clinical Depression and Lack of Awareness of Prediabetes and Diabetes*. For the 594 nonpregnant adults ≥ 20 years who were clinically depressed and had elevated HbA1c values, analyses were then conducted to compare the prevalence of awareness of having HbA1c values in the prediabetes and diabetes ranges according to various sociodemographic characteristics, health-care access or use characteristics, and other factors. We note that none of the adults who indicated that they had been told by a health provider that they had diabetes, had prediabetes, or were borderline for diabetes had HbA1c values in the normal range. As can be seen in Table 3, when extrapolated to the civilian, noninstitutionalized United States population, there were no statistically significant relationships between this awareness prevalence regarding having HbA1c values in the prediabetes and diabetes ranges according to years of NHANES data collection (2007-2008, 2009-2010, and 2011-2012) nor were there statistically significant relationships when considering various characteristics (age groups, race/ethnicity, sex, physical inactivity, and education level). However, these individuals were significantly more likely to be aware of having elevated glucose if they had higher BMI (63.8% had this awareness if obese, 32.1% if overweight, and 13.4% with normal BMI; *P* < 0.001); had a family history of diabetes compared to not having such a history (56.1% versus 38.0%, *P* = 0.004); had a larger number of visits to a doctor in the past year (55.0% if at least 4 such visits, 47.5% if 2-3 visits, and 22.0% if fewer than 2 visits; *P* < 0.001); had health insurance coverage as compared to not having this coverage (53.0% versus 33.9%; *P* = 0.004); had a usual source of care as opposed to not having one (55.2% versus 19.4%; *P* < 0.001); and indicated that they were taking medication for elevated blood pressure and/or elevated cholesterol as compared with indicating that they were not taking such medication (63.9% versus 44.6%; *P* = 0.028).

## 4. Discussion

Among nonpregnant adults, our findings indicate that those with elevated HbA1c levels are significantly more likely than those with normal HbA1c levels to be older, have less formal education, have higher BMI, have a greater family history of diabetes, make a greater number of past year physician visits, be less physically active, and be taking medication for hypertension or hypercholesterolemia. Many of these findings are consistent with other research findings regarding prediabetes and diabetes risk [35–37].

Findings from available studies also show a consistent relationship between depression and diabetes. These findings are further supported by the current study that showed that nonpregnant adults with clinical depression were significantly more likely to have elevated HbA1c readings as compared with those without clinical depression. Persons with elevated glucose that cooccurs with depression are likely to practice poorer self-care and have impaired treatment adherence, worsened glycemic control, and reduced quality of life than those for whom there is no cooccurring depression [38]. Thus, although the proportion of persons with both elevated HbA1c levels and clinical depression is only 6.1% higher than the proportion of adults with elevated HbA1c levels alone, this higher proportion is of considerable concern.

Many of the adults with cooccurring depression were unaware of their elevated glucose readings. This is particularly unfortunate in view of the importance of early identification of prediabetes and diabetes to limit or delay diabetes-related complications among vulnerable populations, such as those with clinical depression. Adults with cooccurring depression who were younger and had lower BMI, no family history of diabetes, no usual source of health care, no health insurance coverage, and few annual visits to health-care providers were especially unlikely to be aware of their elevated glucose readings. Clinicians may view individuals who lack common diabetes risk factors, such as being overweight or being genetically predisposed to diabetes [39], as not needing to be screened for elevated glucose. This may suggest why so many of these individuals were unaware of their high glucose readings. Individuals who have limited contact with health-care providers (often as a result of not having a usual source of care and not having health insurance coverage [40]) may also fail to be screened for prediabetes and diabetes. They may therefore also remain unaware of their out-of-range glucose values, denying them opportunities to proactively address this important health concern.

The large number of adults with clinical depression who are unaware of their elevated glucose levels suggests the importance of finding sites of opportunity for diabetes screening in addition to the doctor's office. In view of the effectiveness of nurse practitioners in supporting patients' needs in diabetes education and health promotion, one such important diabetes screening site is that in which the nurse practitioner has a lead role [41]. Other potential sites for diabetes screening include pharmacies and optometry venues [42, 43]. In addition, each year, many people fail to see a primary care clinician but they do make at least one visit to the dentist [44]. Thus, another potential site for diabetes screening is the dental office. Notably, of the 6,519 noninstitutionalized adults with depression in 16 states who participated in the 2008 Behavioral Risk Factor Surveillance System [45], more than 50 percent indicated that they visited a dental provider in the past year [46]. In fact, there is a growing movement in dentistry to support the identification of metabolic conditions, with many dentists expressing their willingness to incorporate screening for medical conditions in their practices [47, 48]. In our previous research, we showed that the dental visit offers an important alternate site for diabetes screening for patients with periodontal disease [49–51], a diabetes correlate that is common among persons with psychiatric illness [52].

We acknowledge as a limitation of our study the use of various self-report measures, including the PHQ-9. However, we note that this instrument was specifically developed for and is commonly used in primary care settings to screen for depression and is included in the DSM-5 as a recommended severity measure of clinical depression [27, 32, 53–55]. Self-report was also used in reporting on individuals' awareness of elevated glucose readings, family history of diabetes, health access and use measures, health insurance coverage, and the use of medications for elevated blood pressure and elevated cholesterol. Findings should therefore be interpreted with this drawback in mind. We also acknowledge as a limitation of our study the fact that we did not use any of the available risk assessments designed to identify persons at risk for type 2 diabetes. While some of these tools have been found to be useful as clinical tools to identify persons at risk for developing diabetes, the manner in which NHANES's questions were phrased made adaptation of these tools difficult. For example, the Finnish Diabetes Risk Score (FINDRISC) [56] asks about possible diabetes diagnoses of specific categories of persons in the respondent's family (e.g., grandparents, aunts, uncles, and first cousins), and NHANES 2011-2012 did not collect this information. Similarly, the American Diabetes Associations' Diabetes Risk Test asks if the person is physically active [57]. NHANES gathers detailed information about moderate and vigorous physical activity at work or as part of recreational activity, and a person might not respond to these questions in the same way.

In spite of these limitations, our study provides data in strong support of diabetes screening for persons with depression. Further research is needed to examine various alternate sites of opportunity to screen for diabetes in this vulnerable population and to increase their awareness of glucose values. Qualitative research could additionally shed light on the particular experiences of persons with depression and their decision-making processes concerning whether or not to see a primary care provider. Development of more specific treatment approaches based on the individual patient's characteristics and preferences could lead to a more rapid and efficient management of diabetes and depression in clinical practice. In view of the growing number of people with unrecognized prediabetes and diabetes and the increased risk for diabetes among patients with depression, finding alternate sites of opportunity for diabetes screening for these individuals presents an important public health opportunity.

## Figures and Tables

**Table 1:** Characteristics of nonpregnant persons ≥20 years of age according to HbA1c values in the prediabetes/diabetes ranges (in %)^1^.

Characteristic	HbA1c < 5.7% (39 mmol/mol)	HbA1c ≥ 5.7% (39 mmol/mol)	Significance
Age			
20–44	57.0	22.0	<0.001
45–64	37.6	59.5	
≥65	5.5	18.5	
Race/ethnicity			
White, non-Hispanic	66.0	59.0	0.084
Black, non-Hispanic	12.3	17.0	
Mexican American	8.5	8.3	
Other	18.2	15.7	
Sex			
Men	35.7	35.3	0.910
Women	64.3	64.7	
Education			
<high school	25.6	36.7	0.002
High school	24.0	29.4	
>high school	50.4	33.9	
BMI			
Normal (<25 kg/m^2^)	31.8	17.9	<0.001
Overweight (≥25 & <30 kg/m^2^)	33.1	21.2	
Obese (≥30 kg/m^2^)	35.1	60.9	
Family history of diabetes			
No	56.2	45.2	0.004
Yes	43.8	54.8	
Number of doctor visits in the past year			
<2	25.8	16.9	<0.001
2-3	21.7	17.1	
≥4	52.5	65.9	
Health coverage status			
Not covered	29.0	24.9	0.229
Covered	71.0	75.1	
Usual source of care			
Clinic or doctor's office	77.4	81.0	0.265
Other or none	22.6	19.0	
Physical inactivity^2^			
No	55.5	36.9	<0.001
Yes	44.5	63.1	
Takes medications for hypertension/hypercholesterolemia			
No	80.6	47.2	<0.001
Yes	19.4	52.8	

^1^When extrapolated to the civilian, noninstitutionalized population in the United States of nonpregnant persons, ≥20 years.

^2^A person is recorded as physically inactive if he/she indicated spending <30 minutes of moderate or vigorous activity at work and/or during leisure time each day.

**Table 2:** Relationship between clinical depression (as measured by the PHQ-9) and HbA1c measures for the nonpregnant United States population ≥20 years of age (in %).

	Not clinically depressed (PHQ-9 < 10)	Clinically depressed (PHQ-9 ≥ 10)
HbA1c < 5.7% (39 mmol/mol)	67.7	61.6
HbA1c ≥ 5.7% (39 mmol/mol)	32.3	38.4

**Table 3:** Prevalence of awareness of having HbA1c values in the prediabetes/diabetes ranges in nonpregnant persons ≥20 years of age with clinical depression and elevated HbA1c (in %).

Characteristic	Persons with HbA1c ≥ 5.7% (39 mmol/mol)	Persons aware that they have diabetes or prediabetes	% aware that they have diabetes or prediabetes^1^	95% confidence interval^1^	Sig.
Year					
2007-2008	200	95	46.5	(35.3, 58.1)	0.636
2009-2010	213	99	45.2	(35.7, 55.0)	
2011-2012	181	103	52.6	(38.2, 66.6)	
Age					
20–44	127	45	37.8	(28.4, 48.1)	0.081
45–64	324	171	50.6	(40.8, 60.3)	
≥65	143	81	52.8	(42.7, 62.6)	
Race/ethnicity					
White, non-Hispanic	235	117	50.5	(40.8, 60.1)	0.381
Black, non-Hispanic	140	75	49.4	(39.0, 59.8)	
Mexican American	98	48	46.1	(32.1, 60.9)	
Other	121	57	39.2	(28.6, 50.9)	
Sex					
Men	212	102	43.2	(34.8, 51.9)	0.165
Women	382	195	50.9	(42.1, 59.1)	
Education					
<high school	271	143	50.6	(42.0, 59.1)	0.727
High school	140	66	48.3	(37.4, 59.5)	
>high school	183	88	45.4	(34.0, 57.3)	
BMI					
Normal (<25 kg/m^2^)	89	21	13.4	(7.4, 22.9)	<0.001
Overweight (≥25 & <30 kg/m^2^)	128	45	32.1	(19.0, 48.9)	
Obese (≥30 kg/m^2^)	367	225	63.8	(55.7, 71.3)	
Family history of diabetes					
No	244	96	38.0	(29.3, 47.6)	0.004
Yes	334	194	56.1	(47.5, 64.3)	
Number of doctor visits in the past year					
<2	94	22	22.0	(11.9, 37.2)	<0.001
2-3	111	55	47.5	(33.8, 61.7)	
≥4	389	220	55.0	(47.4, 62.5)	
Health coverage status					
Not covered	157	64	33.9	(23.8, 45.7)	0.004
Covered	437	233	53.0	(45.4, 60.5)	
Usual source of care					
Clinic or doctor's office	480	261	55.2	(48.4, 61.9)	<0.001
Other or none	103	29	19.4	(10.7, 32.6)	
Physical inactivity^2^					
No	211	104	44.9	(35.8, 54.4)	0.335
Yes	383	193	50.1	(41.9, 58.2)	
Takes medications for hypertension/hypercholesterolemia					
No	476	223	44.6	(37.1, 52.4)	0.028
Yes	118	74	63.9	(48.4, 77.0)	

^1^When extrapolated to the civilian, noninstitutionalized population in the United States of nonpregnant persons, 20 years of age or older with clinical depression and elevated HbA1c measures.

^2^A person is recorded as physically inactive if he/she indicated spending <30 minutes of moderate or vigorous activity at work and/or during leisure time each day.
